# Muscle Twitch Kinetics Are Dependent on Muscle Group, Disease State, and Age in Duchenne Muscular Dystrophy Mouse Models

**DOI:** 10.3389/fphys.2020.568909

**Published:** 2020-09-25

**Authors:** Kyra K. Peczkowski, Neha Rastogi, Jeovanna Lowe, Kyle T. Floyd, Eric J. Schultz, Tallib Karaze, Jonathan P. Davis, Jill A. Rafael-Fortney, Paul M. L. Janssen

**Affiliations:** Department of Physiology and Cell Biology, College of Medicine, The Ohio State University, Columbus, OH, United States

**Keywords:** skeletal muscle, muscular dystrophies, contraction, relaxation, age

## Abstract

Duchenne muscular dystrophy (DMD) is an X-linked disorder caused by the lack of functional dystrophin protein. In muscular dystrophy preclinical research, it is pertinent to analyze the force of the muscles affected by the disease to assess pathology and potential effectiveness of therapeutic interventions. Although muscles function at sub-maximal levels *in vivo*, maximal tetanic contractions are most commonly used to assess and report muscle function in muscular dystrophy studies. At submaximal activation, the kinetics of contraction and relaxation are heavily impacted by the kinetics of the single twitch. However, maximal tetanic force is often the main, if not sole, outcome measured in most studies, while contractile kinetics are rarely reported. To investigate the effect of muscle disease on twitch contraction kinetics, isolated diaphragm and *extensor digitorum longus* (EDL) muscles of 10-, 20-week, “het” (dystrophin deficient and utrophin haplo-insufficient), and 52-week *mdx* (dystrophin deficient) mice were analyzed and compared to wild-type controls. We observed that twitch contractile kinetics are dependent on muscle type, age, and disease state. Specific findings include that diaphragm from wildtype mice has a greater time to 50% relaxation (RT50) than time to peak tension (TTP) compared to the het and *mdx* dystrophic models, where there is a similar TTP compared to RT50. Diaphragm twitch kinetics remain virtually unchanged with age, while the EDL from het and *mdx* mice initially has a greater RT50 than TTP, but the TTP increases with age. The difference between EDL contractile kinetics of dystrophic and wildtype mice is more prominent at young age. Differences in kinetics yielded greater statistical significance compared to previously published force measurements, thus, using kinetics as an outcome parameter could potentially allow for use of smaller experimental groups in future study designs. Although this study focused on DMD models, our findings may be applicable to other skeletal muscle conditions and diseases.

## Introduction

Duchenne muscular dystrophy (DMD) is a degenerative muscle disease that primarily affects male children (Hoffman et al., 1987; Koenig et al., 1987). DMD is an X-linked disorder caused by a mutation in the DMD gene (Hoffman et al., 1987), and it affects approximately 1 in 3,500–5,000 males born (Emery, 1991; Mah et al., 2014). The mutation results in the partial or total loss of the functional dystrophin protein (Monaco et al., 1988; Koenig et al., 1989) leading to membrane disruption and muscle weakness. The symptoms of Duchenne muscular dystrophy typically onset in young males between the ages of 3 and 5 years, with initial symptoms including delay in motor function, abnormal stride, difficulty standing from a sitting position and maintaining balance (Darras et al., 2015). As the disease worsens, it affects all striated muscles, including weakening of the diaphragm (Khirani et al., 2014; Gudmundson et al., 2019; Pennati et al., 2019) and limb muscles (McDonald et al., 1995; Wallace and McNally, 2009), and in later stages, the disease also affects the heart (Buddhe et al., 2018; Mavrogeni et al., 2018; Meyers and Townsend, 2019).

In muscular dystrophy research, it is often pertinent to analyze the developed force of the muscles affected by the disease to assess pathology and potential efficacy of interventions. Widely used mouse models for DMD preclinical studies include the dystrophin deficient *mdx* mouse (Bulfield et al., 1984; Yucel et al., 2018) and the dystrophin deficient and utrophin haplo-insufficient (*utrn*^+/–^; *mdx*) “het” mouse (Zhou et al., 2008; van Putten et al., 2012). Maximal tetanic contractions are the most commonly used protocol to assess muscle function (Hibaoui et al., 2011; Janssen et al., 2014; Lowe et al., 2016, 2018; Hauck et al., 2019; Lindsay et al., 2019; Trajanovska et al., 2019). However, *in vivo*, the vast majority of muscle function occurs not at maximal, but at sub-maximal contraction conditions. At these physiologically relevant sub-maximal conditions, the kinetics of contraction and relaxation play an important role. When a muscle is slow to relax, due to slow kinetics of the force-generating contractile system, forces of the antagonist muscle of a given joint can impose additional stress on the agonist muscle. This situation would lead to more muscle damage, as this antagonist stress results in eccentric stress on the agonist, and this eccentric stress is known to exacerbate muscle damage (Proske and Morgan, 2001). Therefore, analysis of the kinetics of contraction and relaxation could further our understanding of the contractile deficiencies that are apparent in DMD, and related neuromuscular diseases. Here, we investigated the effect of muscular dystrophy on twitch contraction kinetics, in two distinctly different muscle groups, isolated diaphragm and *extensor digitorum longus* (EDL) muscles of het, *mdx*, and wild-type C57BL/10 mice, as well as how these parameters are affected by age.

## Materials and Methods

### Mice

This study was a retrospective analysis of the kinetics of diaphragm and EDL muscles used for maximal tetanic forces in previously published manuscripts (Lowe et al., 2015, 2016, 2018). As in previous studies conducted in our laboratory, male dystrophin-deficient, utrophin haplo-insufficient (utrn ^+/–^; mdx) “het” mice and dystrophin-deficient mdx mice were bred and genotyped as previously described (Lowe et al., 2015, 2016, 2018). The het and mdx mice were used for treatment groups or untreated controls. Male C57BL/10 were bred as previously described and used as wildtype controls.

### *In vivo* Diaphragm and Extensor Digitorum Longus (EDL) Contraction Force Measurements

Mice were heparinized, and 5–10 min later were sacrificed by cervical dislocation followed by rapid removal of the heart. The ribcage and diaphragm were removed and placed in a modified Krebs-Henseleit solution [95% O_2_/ 5% CO_2_ (pH 7.4), 5 mM KCl, 127 mM NaCl, 1.2 mM NaH_2_PO_4_, 1.2 mM MgSO_4_, 20 mM NaHCO_3_, 10 mM D-glucose, and 0.25 mM CaCl_2_] with 20 mM 2,3-butanedione monoxime (BDM) to prevent muscle damage during dissection. Two linear strips of diaphragm, about 3 mm in width, were cut from the center of the diaphragm along with the connective tissue and ribcage, which allowed the muscle to be securely mounted inside a bath with circulating Krebs-Henseleit solution (pH 7.4, 95% O_2_/ 5% CO_2_). The temperature was maintained at 37°C (Murray et al., 2012). Strips were placed in an experimental chamber between two parallel electrodes, and attached to a KG2 force transducer (World Precision Instruments) on one side and a linear servo-driven micromanipulator on the opposite side. Twitch contractions were elicited by a single 1 ms pulse. The muscle was slowly stretched by a few percentage of its’ length, and after 1–2 min of rest another twitch was assessed. This was repeated until optimal length of the muscle was achieved, reflected by optimal force development, and no further increase upon the next stretch. Force and kinetic data were recorded during the protocol with a custom computer program written in LabView.

The EDL muscle from each leg was excised under a dissection microscope and kept in modified Krebs-Henseleit solution with BDM until force measurements. The forces of both right and left EDLs were recorded for each mouse. Silk sutures were secured on the upper and lower tendons of the muscles in order to mount the muscle in the experimental set-up equipped with a KG7 force transducer (World Precision Instruments). The temperature of the circulating Krebs-Henseleit solution (pH 7.4, 95% O_2_/ 5% CO_2_) was maintained at 30°C (Janssen et al., 2014). Optimal length was determined by slowly increasing the length of the muscle via a micromanipulator and subjecting the muscle to a single twitch contraction. Optimal length was determined when the active developed force no longer increased, and remained constant for two subsequent contractions. Force and kinetic data (time to peak tension, and time from peak tension to 50% relaxation) was recorded during the protocol with a custom-written program in LabView.

The kinetics for the diaphragm and EDL muscles were analyzed by custom analysis software written in LabVIEW. Active developed twitch tension, time to peak tension (TTP), and time to 50% relaxation (RT50) were assessed from the single twitch contraction at optimal length of individual EDL and diaphragm muscles. Raw data was analyzed for significance with unparalleled two-sided *t*-tests using GraphPad Prism.

The temperature of the diaphragm (37°C) and the EDL (30°C) were equal in all 3 studies (Lowe et al., 2015, 2016, 2018). The diaphragm temperature is that of core murine body temperature and has been shown to be reliable and reproducible at this temperature (Murray et al., 2012). The EDL temperature is lower due to being a limb muscle but is higher than average room temperature. Since kinetics are slower at room temperature compared to physiological temperatures (Hill, 1972), we chose to conduct these past experiments at the temperature that was most relevant physiologically.

The protocols for the three previous studies were conducted the same. Force measurements were performed by individuals blinded to the genotype of the mice. Within each study, the force measurements were performed by the same individual to limit variability.

## Results

The specific goal of this study was to determine if disease state impacts the kinetics of skeletal muscle twitch contractions, in different models, in different muscle groups, and at different ages, using Duchenne muscular dystrophy as the disease model. The mice used in this retrospective studies were 10 weeks (Lowe et al., 2015), 20 weeks (Lowe et al., 2016) and 52 weeks of age (Lowe et al., 2018). The genotypes of the mice were wild-type C57BL/10 (C57), utrophin haplo-insufficient “het,” and dystrophin-deficient *mdx*. Time to peak and time for 50% relaxation was determined for submaximal twitches of the diaphragm and EDL at optimal length.

**FIGURE 1:**
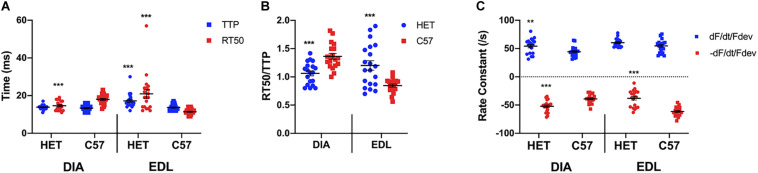
**(A)** Time to peak and 50% relaxation of 10 week old dystrophic and wildtype muscle types. Kinetic analysis was conducted in diaphragm and EDL muscles from the same mice. Mouse models include dystrophic HET and wildtype C57BL/10. Statistical analysis was conducted using a two-sided *t*-test. *** indicates *P* < 0.001 compared to C57BL/10 mice. The line represents the group mean; error bars are standard error of mean. **(B)** Ratio of time to 50% relaxation and time to peak (RT50/TTP). Mouse models include dystrophic HET and wildtype C57BL/10. Statistical analysis was conducted using a two-sided *t*-test.*** indicates *P* < 0.001 compared to C57BL/10 mice. The line represents the group mean; error bars are standard error of mean. **(C)** Rate constants of individual diaphragm and EDL twitches. Mouse models include dystrophic HET and wildtype C57BL/10. Statistical analysis was conducted using a two-sided *t*-test. ** indicates *P* < 0.01, *** indicates *P* < 0.001 compared to C57BL/10 mice. The line represents the group mean; error bars are standard error of mean.

As shown in Figure 1A, a two-sided *t*-test indicated a significant difference was not present between the diaphragm TTP of 10 week het mice (13.8 ± 0.3 ms, *n* = 19) and their C57 counterparts (13.3 ± 0.3 ms; *n* = 19; *p* = 0.2478). However, there was a significant difference between the diaphragm RT50 of the 10-week het mice (14.6 ± 0.6 ms; *n* = 19) and the C57 controls (18.0 ± 0.6 ms; *n* = 19; *p* = 0.0002), with the het mice relaxing quicker than the wildtype mice. With the EDL kinetics, both the TTP (17.4 ± 0.8 ms; *n* = 20) and RT50 (21.0 ± 2.3 ms; *n* = 20) of the het EDL muscles were significantly prolonged compared to the TTP (13.7 ± 0.3 ms; *n* = 20; *p* = 0.0002) and RT50 (11.5 ± 0.3 ms; *n* = 20; *p* = 0.0002) of the C57 mice. In addition, the het EDL muscles took a longer time period to relax than to contract, differing from the C57 muscles. Figure 1B shows the balance between time to peak tension and time to 50% relaxation (RT50/TTP). The ratio for both het diaphragm and EDL was significantly different compared to the C57 mice of the same age. The ratio for the het diaphragms (1.1 ± 0.0; *n* = 19) was significantly lower than the wildtype diaphragms (1.4 ± 0.1; *n* = 19, *p* < 0.0001). Contrary to the diaphragms, the ratio for het EDLs (1.2 + 0.1; *n* = 20) was significantly greater than the wildtype EDLs (0.8 + 0.0; *n* = 20; *p* = 0.0001). As depicted in Figure 1C, the positive rate constant of the het diaphragms (54.2 ± 3.1/s; *n* = 19; *p* = 0.0083) was significantly greater compared to the positive rate constant of the C57 diaphragms (43.1 ± 1.7/s; *n* = 19). The negative rate constant of the het diaphragms (-52.3 ± 2.4/s; *n* = 19; *p* < 0.0001) was also significantly greater compared to that of the C57 diaphragms (-39.1 ± 1.8/s; *n* = 19). Only the negative rate constant of the het EDLs were significantly different compared to the C57 EDLs. There was no significance between the positive rate constant of the het EDLs (60.4 ± 1.6/s; *n* = 20; *p* = 0.08) compared to the C57 EDLs (54.8 ± 2.6/s; *n* = 20). The het (-38.1 ± 3.2/s; *n* = 20; *p* < 0.0001) negative rate constant was significantly lower than that of the C57 EDLs (-61.3 ± 1.8/s; *n* = 20).

**FIGURE 2:**
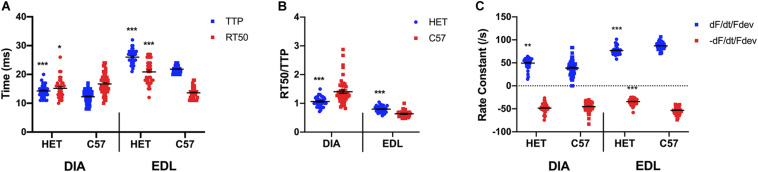
**(A)** Time to peak and 50% relaxation of 20 week old dystrophic and wildtype muscle types. Kinetic analysis was conducted in diaphragm and EDL muscles from the same mice. Mouse models include dystrophic HET and wildtype C57BL/10. Statistical analysis was conducted using a two-sided *t*-test. * indicates *P* < 0.05, ** indicates *P* < 0.01, and *** indicates *P* < 0.001 compared to C57BL/10 mice. The line represents the group mean; error bars are standard error of mean. **(B)** Ratio of time to 50% relaxation and time to peak (RT50/TTP). Mouse models include dystrophic HET and wildtype C57BL/10. Statistical analysis was conducted using a two-sided *t*-test. *** indicates *P* < 0.001 compared to C57BL/10 mice. The line represents the group mean; error bars are standard error of mean. **(C)** Rate constants of individual diaphragm and EDL twitches. Mouse models include dystrophic HET and wildtype C57BL/10. Statistical analysis was conducted using a two-sided *t*-test. ** indicates *P* < 0.01, *** indicates *P* < 0.001 compared to C57BL/10 mice. The line represents the group mean; error bars are standard error of mean.

As shown in Figure 2A, a two-sided *t*-test indicated that a significant difference is present between TTP of the 20 week old het mice (14.3 ± 0.4 ms; *n* = 34) diaphragm and the C57 mice (12.3 ± 0.3 ms; *n* = 51; *p* < 0.0001), with the het muscles having a longer TTP. A significant difference was present between the RT50 of the het (15.2 ± 0.6 ms; *n* = 34) and C57 (16.7 ± 0.5 ms; *n* = 51; *p* = 0.04951) diaphragms. A significant difference was also indicated in both the TTP (24.9 ± 1.0 ms; *n* = 38) and RT50 (20.9 ± 0.7 ms; *n* = 34) of the het EDL muscles compared to the TTP (21.1 ± 0.9 ms; *n* = 39; *p* = 0.005) and RT50 (13.6 ± 0.3 ms; *n* = 35; *p* < 0.0001) of the C57 EDL muscles, with both contraction and relaxation being prolonged. EDL muscles contracted at a slower rate than they relaxed in both C57 and het mice, whereas the diaphragm relaxed at a slower rate than it contracted in both mouse models. As shown in Figure 2B, the kinetics ratio of het diaphragms (1.1 ± 0.0; *n* = 34) was significantly lower than the C57 diaphragms (1.4 ± 0.1; *n* = 51; *p* < 0.0001). The ratio for het EDLs (0.8 ± 0.2; *n* = 34) was significantly greater than the ratio of wildtype EDL (0.6 ± 0.0; *n* = 35; *p* < 0.0001) kinetics. The RT50/TTP ratio of the het diaphragms remained unchanged between 10 and 20 weeks of age, whereas the ratio for het EDLs decreased between 10 and 20 weeks of age. As shown in Figure 2C, the positive rate constant of the het diaphragms (49.5 ± 2.1/s; *n* = 34; *p* = 0.002) was significantly greater than the C57 diaphragms (38.6 ± 2.4/s; *n* = 51) at 20 weeks. No significant difference was present in the negative rate constant of the het diaphragms (-48.3 ± 1.8/s; *n* = 34; *p* = 0.2) compared to the C57 diaphragms (-45.2 ± 1.4/s; *n* = 51) at this age. Significant differences were present in the positive and negative rate constants of the het EDLs compared to the C57 EDLs at 20 weeks. The positive rate constant of the het EDLs (76.9 ± 1.5/s; *n* = 34; *p* < 0.0001) was significantly lower than the C57 EDLs (87.1 ± 1.3/s; *n* = 35). A similar pattern was seen in the negative rate constant, as the het EDLs (-34.2 ± 1.2/s; *n* = 34; *p* < 0.0001) had a lower negative rate constant compared to the C57 EDLs (-53.3 ± 1.4/s; *n* = 35).

**FIGURE 3:**
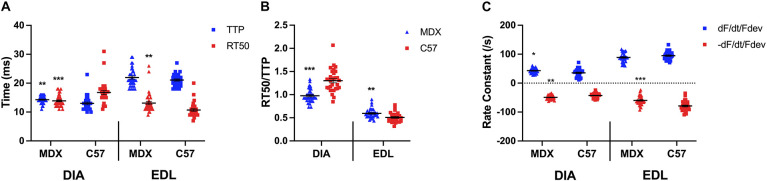
**(A)** Time to peak and 50% relaxation of 52 week old dystrophic and wildtype muscle types. Kinetic analysis was conducted in diaphragm and EDL muscles from the same mice. Mouse models include dystrophic MDX and wildtype C57BL/10. Statistical analysis was conducted using a two-sided *t*-test. ** *P* < 0.01 and *** *P* < 0.001 compared to C57BL/10 mice. The line represents the group mean; error bars are standard error of mean. **(B)** 50% relaxation of 52 week old dystrophic and wildtype muscle types. Mouse models include dystrophic MDX and wildtype C57BL/10. Statistical analysis was conducted using a two-sided *t*-test. ** *P* < 0.01 and *** *P* < 0.001 compared to C57BL/10 mice. The line represents the group mean; error bars are standard error of mean. **(C)** Rate constants of individual diaphragm and EDL twitches. Mouse models include dystrophic MDX and wildtype C57BL/10. Statistical analysis was conducted using a two-sided *t*-test. * *P* < 0.05, ** *P* < 0.01, and *** *P* < 0.001 compared to C57BL/10 mice. The line represents the group mean; error bars are standard error of mean.

As indicated by a two-sided *t*-test in Figure 3A, the TTP of diaphragm muscles of 52 week old *mdx* mice (14.3 ± 0.2 ms; *n* = 35) was significantly prolonged compared to the TTP of C57 (13.0 ± 0.5 ms; *n* = 30; *p* = 0.0095) diaphragms, whereas the RT50 of *mdx* (13.9 ± 0.3 ms; *n* = 35) diaphragms had a faster relaxation compared to the wildtype mice (16.8 ± 0.7 ms; *n* = 30; *p* = 0.0004). However, the TTP of the *mdx* (21.9 ± 0.5 ms; *n* = 35) and C57 (21.1 ± 0.4 ms; *n* = 30; *p* = 0.1998) EDL muscles were not significantly different. The RT50 (13.1 ± 0.6 ms; *n* = 35) of the *mdx* EDL muscles were significantly prolonged compared to the RT50 of the C57 (10.7 ± 0.5 ms; *n* = 30; *p* = 0.0026) mouse model. Similar to the het mouse model, old *mdx* EDL muscles contract at a slower rate than they relax. Figure 3B shows the kinetics ratio of diaphragms from dystrophic (*mdx*) mice (1.0 ± 0.0; *n* = 35) remains to be significantly lower compared to diaphragms from C57 mice (1.3 ± 0.0; *n* = 30; *p* < 0.0001) at 52 weeks of age. The ratio of EDLs from dystrophic (*mdx*) mice (0.6 ± 0.0; *n* = 35) at 52 weeks of age remains significantly greater than the kinetics ratio of EDL from C57 mice (0.5 ± 0.0; *n* = 30; *p* = 0.001), however, the ratios become more similar with age and the significance decreases. As shown in Figure 3C, there is a significant difference in positive and negative rate constants in diaphragms from 52 week old *mdx* mice compared to C57 mice. The positive rate constant of the *mdx* diaphragms (43.0 ± 1.4/s; *n* = 35; *p* = 0.01) was significantly greater than the C57 diaphragms (35.6 ± 2.6/s; *n* = 30) the same holds true for the negative rate constant of the *mdx* diaphragms (-49.3 ± 1.2/s; *n* = 35; *p* = 0.001) compared to the C57 diaphragms (-42.8 ± 1.5/s; *n* = 30). There was no significant difference between the positive rate constant of the *mdx* EDLs (89.0 ± 2.6/s; *n* = 35; *p* = 0.1) and the C57 EDLs (94.9 ± 2.5/s; *n* = 30) at this age. The negative rate constant of the *mdx* EDLs (-59.7 ± 2.5/s; *n* = 35; *p* < 0.0001) was significantly lower than that of the C57 EDLs (-78.9 ± 3.1/s; *n* = 30).

**FIGURE 4:**
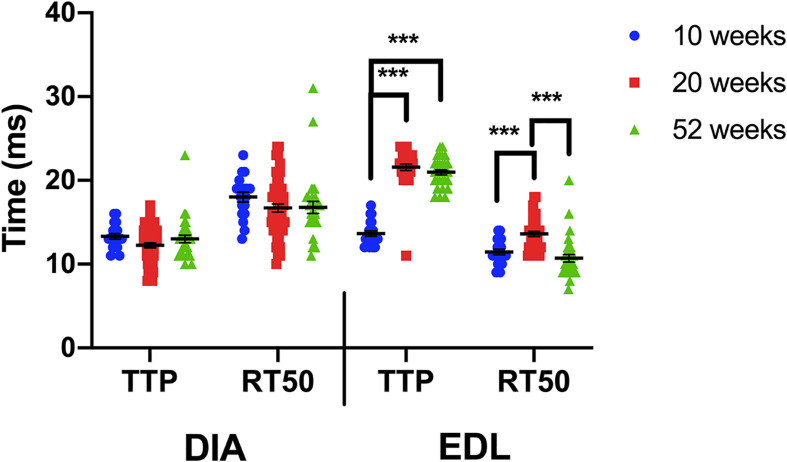
Time to peak and 50% relaxation of wildtype diaphragm and EDL muscles at 10, 20, and 52 weeks of age. Kinetic analysis was conducted in diaphragm and EDL muscles of C57BL/10 mice. The data shown in this figure is the same as in previous figures but is formatted in a manner to compare kinetics with increasing age. Statistical analysis was conducted using a one-way ANOVA. *** indicates *P* < 0.001. The line represents the group mean; error bars are standard error of mean.

When the kinetics for each muscle type are compared between the 3 age groups, a one-way ANOVA indicates that the kinetics of the diaphragm are not age-dependent, whereas the EDL is influenced by age (Figure 4). The diaphragm kinetics remain virtually unchanged at 10, 20, and 52 weeks of age. The largest change in kinetics occurs between 10 and 20 weeks of age in the EDL.

## Discussion

The majority of muscle function *in vivo* occurs at the sub-maximal level. At this sub-maximal force level, the amount of force generated depends on both the stimulation frequency, and the kinetics of muscle contraction and relaxation. There are several studies that include kinetic data in preclinical muscular dystrophy studies, however, these studies were not conducted at physiological temperatures and/or investigated one muscle type of a single dystrophic mouse model (Anderson et al., 1988; Quinlan et al., 1992; Hayes et al., 1993; Lynch et al., 1997; Hayes and Williams, 1998; Chan and Head, 2010; Kiriaev et al., 2018; Addinsall et al., 2020). Although maximal force development is very similar at room and body temperature (Hill, 1972), temperature is a critically important component of kinetics studies; kinetics of contraction and relaxation are much slower at room temperature compared to physiological temperature. The diaphragm is located in the core of the body which is maintained at ∼37°C. Limbs are typically maintained at a lower temperature than the body’s core. The average temperature of the lower extremities (i.e., shins) is between 29 and 30°C, which is virtually the same as the upper extremities (i.e., palms) (Gatt et al., 2015). The diaphragm and EDL protocols were thus both conducted at temperatures that reflect their individual physiological temperatures (37 and 30°C, respectively).

The majority of previous reports specifically focused on maximum tetanic tension. Maximal tetanic tension is a level of muscle activation that is not commonly reached *in vivo*, as near-maximal force only occurs during pathological situations, like severe muscle cramping. Our results show that diaphragm and EDL twitch muscle kinetics are significantly affected in models of muscular dystrophy and are affected differently by both disease state and age.

The analysis of kinetics of dystrophic mouse models versus WT mice suggested that diaphragm muscles and EDL muscles behave differently, both in inherent property of the kinetics, as well as the impact disease has on these kinetics. In general, diaphragm muscle contracts at a faster rate than the muscles relaxes. However, EDL muscles contract at a slower rate than they relax, except at 10 weeks of age. In addition, the rate of contraction and relaxation of the diaphragm is not dependent on age in C57 mice, whereas the results suggest that the rate of relaxation and contraction is dependent on age in EDL muscles.

Differences in muscle fiber types between the diaphragm and EDL could contribute to the muscle behavior reported in this study. Contrary to the human diaphragm, rat and mouse diaphragms are fast muscle, consisting predominantly of type IIX fibers. The EDL is also a fast muscle, predominantly consisting of type IIB fibers (Schiaffino et al., 1989; Schiaffino and Reggiani, 2011). Type IIB fibers are the fastest fiber type to contract but also the fastest to fatigue. Fast muscle fibers are preferentially affected in Duchenne muscular dystrophy (Webster et al., 1988), with type IIX being the first to degenerate in humans (Pedemonte et al., 1999). The degeneration and alteration of muscle fiber types was not focused on in this study. It is of interest to investigate the proportions of muscle fiber types in dystrophic mice at various ages and the implications this may have on contractile kinetics in Duchenne muscular dystrophy.

The statistical variability in kinetics is much smaller than that of the force of skeletal muscle. The level of force is dependent on several variables that need to be measured, such as cross-sectional area, and which lead to a significant larger variability in the data than timing kinetics, that are not subjected to the size of the muscle. This lower degree of variability in the kinetics data would allow for a significant reduction in the number of animal subjects needed to reach statistical significance. In our own past studies, when maximal developed force is the primary outcome measure of physiology studies, power analysis dictated *n* = 18 animals per group (Lowe et al., 2016, 2018). However, using contractile kinetics (RT50/TTP) as an outcome measure, power analysis using the same parameters indicated as few as 4–7 mice sufficient in most cases with the maximum number of mice being 16, depending on the disease state, age, and muscle type being investigated (Supplementary Material).

A limitation of the present study is that the C57BL/10 mice were maintained as a separate inbred colony for a long period of time, as is typical for most studies involving comparisons to dystrophic animals. Since mouse breeding was optimized to generate het and *mdx* littermates for various published studies and also to obtain large enough cohorts of dystrophic animals to include untreated and treated groups for the manuscripts from which the data for this study were originally generated (Rafael-Fortney et al., 2011; Lowe et al., 2015, 2016, 2018), it was not technically feasible to also produce wild-type littermates for either of these dystrophic genotypes (Bellinger et al., 2009). It is possible that inbreeding has led to additional mutations in the C57BL/10 line that may affect the kinetics of skeletal muscle function. Future studies comparing therapeutic strategies in dystrophic skeletal muscles will be useful for investigating a restoration of “wild-type” function and providing further validation for the innovative findings in the current study.

## Data Availability Statement

The raw data supporting the conclusions of this article will be made available by the authors, without undue reservation.

## Ethics Statement

The animal study was reviewed and approved by Ohio State University Animal Care and Use Committee.

## Author Contributions

KP conducted EDL measurements and wrote the manuscript. NR conducted diaphragm force measurements. JL managed the retrospective studies and performed analyzed data. KF conducted EDL contraction. ES and TK conducted diaphragm measurements. PJ and JR-F designed the study, and edited the manuscript. JD performed data analysis and interpretation. All authors contributed to the article and approved the submitted version.

## Conflict of Interest

The authors declare that the research was conducted in the absence of any commercial or financial relationships that could be construed as a potential conflict of interest.
