# Corrigendum: Muscle Twitch Kinetics Are Dependent on Muscle Group, Disease State, and Age in Duchenne Muscular Dystrophy Mouse Models

**DOI:** 10.3389/fphys.2021.820245

**Published:** 2022-01-05

**Authors:** Kyra K. Peczkowski, Neha Rastogi, Jeovanna Lowe, Kyle T. Floyd, Eric J. Schultz, Tallib Karaze, Jonathan P. Davis, Jill A. Rafael-Fortney, Paul M. L. Janssen

**Affiliations:** Department of Physiology and Cell Biology, College of Medicine, The Ohio State University, Columbus, OH, United States

**Keywords:** skeletal muscle, muscular dystrophies, contraction, relaxation, age

**Error in Figure/Table**

In the original article, there was a mistake in Figure 3 as published. In Figure 3, the C57 EDL RT50% data (Figure 3A, dataset 8) was erroneously duplicated from the C57 DIA RT50% data (Figure 3A, dataset 4). This mistake happened when we re-made our figures for the final submission. The data in the text, as well as statistics, all outcomes, and all conclusions, are all based on the correct data. The corrected Figure 3 appears below.

**Figure 3:**
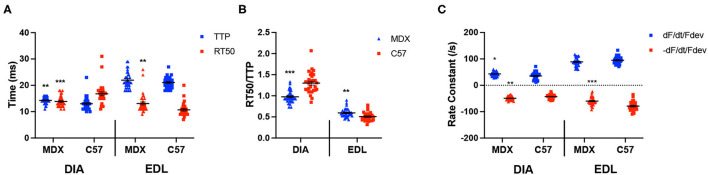
**(A)** Time to peak and 50% relaxation of 52 week old dystrophic and wildtype muscle types. Kinetic analysis was conducted in diaphragm and EDL muscles from the same mice. Mouse models include dystrophic MDX and wildtype C57BL/10. Statistical analysis was conducted using a two-sided *t*-test. ^**^P < 0.01 and ^***^P < 0.001 compared to C57BL/10 mice. The line represents the group mean; error bars are standard error of mean. **(B)** 50% relaxation of 52 week old dystrophic and wildtype muscle types. Mouse models include dystrophic MDX and wildtype C57BL/10. Statistical analysis was conducted using a two-sided *t*-test. ^**^P < 0.01 and ^***^P < 0.001 compared to C57BL/10 mice. The line represents the group mean; error bars are standard error of mean. **(C)** Rate constants of individual diaphragm and EDL twitches. Mouse models include dystrophic MDX and wildtype C57BL/10. Statistical analysis was conducted using a two-sided *t*-test. ^*^P < 0.05, ^**^P < 0.01, and ^***^P < 0.001 compared to C57BL/10 mice. The line represents the group mean; error bars are standard error of mean.

The authors apologize for this error and state that this does not change the scientific conclusions of the article in any way. The original article has been updated.

## Publisher's Note

All claims expressed in this article are solely those of the authors and do not necessarily represent those of their affiliated organizations, or those of the publisher, the editors and the reviewers. Any product that may be evaluated in this article, or claim that may be made by its manufacturer, is not guaranteed or endorsed by the publisher.

