# Supplementary material for: Muscle Twitch Kinetics Are Dependent on Muscle Group, Disease State, and Age in Duchenne Muscular Dystrophy Mouse Models
Source: Front Physiol. 2020 Sep 25;11:568909. doi: 10.3389/fphys.2020.568909 (PMC7545010; doi:10.3389/fphys.2020.568909)
Supplement: Supplementary file 1 [file Data_Sheet_1.pdf]

Supplementary Figure 1. Raw traces of EDL twitches from dystrophic and wildtype mice at 10, 20, and 52 weeks of age. Figure 1A. 10 week HET mouse. **Figure 1B.** 10 week C57BL/10 mouse. **Figure 1C.** 20 week HET mouse. **Figure 1D.** 20 week C57BL/10 mouse. **Figure 1E.** 52 week MDX mouse. **Figure 1F.** 52 week C57BL/10 mouse. Twitches are representative of the groups' mean force and kinetics.

Supplementary Table 1. Power analysis details to determine number of mice required for significance in kinetics studies. A power analysis was conducted for each muscle type (diaphragm and EDL) at each age group (10, 20, 52 weeks) using RT50/TTP and the average standard deviation between dystrophic (HET or MDX) mice and C57BL/10 mice. α= 0.05, power =0.8.

Supplementary Figure 1

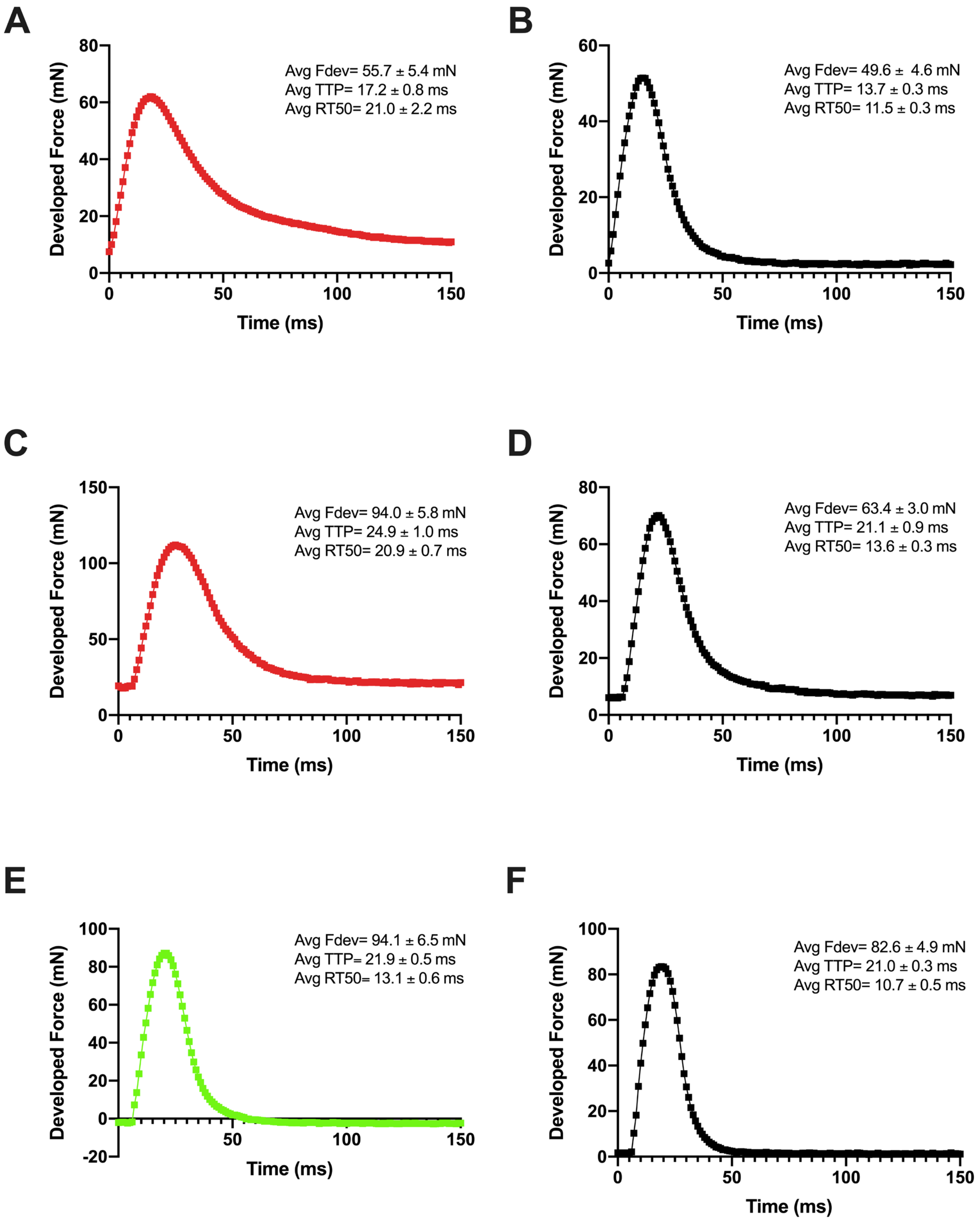

Supplementary Table 1

**Age & Muscle Type**	**µ1**	**µ2**	**Average Standard Deviation**	**N**
10 week Dia	1.1	1.4	0.2	7
10 week EDL	1.2	0.9	0.2	7
20 week Dia	1.1	1.4	0.3	16
20 week EDL	0.8	0.6	0.1	4
52 week Dia	1.0	1.3	0.2	7
52 week EDL	0.6	0.5	0.1	16
